# Effects of Electroacupuncture on the Daily Rhythmicity of Intestinal Movement and Circadian Rhythmicity of Colonic* Per2* Expression in Rats with Spinal Cord Injury

**DOI:** 10.1155/2016/9860281

**Published:** 2016-11-24

**Authors:** Jie Cheng, Xueqiang Wang, Jiabao Guo, Yujie Yang, Wenyi Zhang, Bin Xie, Zhaojin Zhu, Yuemei Lu, Yi Zhu

**Affiliations:** ^1^Nanjing University of Chinese Medicine, Nanjing 210023, China; ^2^Department of Sport Rehabilitation, Shanghai University of Sport, Shanghai 200438, China; ^3^Rehabilitation Medicine Center, The First Affiliated Hospital of Nanjing Medical University, Nanjing 210029, China; ^4^Rehabilitation Center, Hainan Provincial Nongken General Hospital, Haikou 570311, China

## Abstract

*Background*. Spinal cord injury (SCI) leads to bowel dysfunction. Electroacupuncture (EA) may improve bowel function.* Objective*. To assess EA on daily rhythmicity of intestinal movement and circadian rhythmicity of colonic* Per2* expression in rats with SCI.* Methods*. Rats were randomized to the sham, SCI, and SCI+EA groups. EA was performed at bilateral Zusanli point (ST36) during daytime (11:00–11:30) for 14 days following SCI. Intestinal transit and daily rhythmicity of intestinal movement were assessed. Circadian rhythmicity of colonic* Per2* expression was assessed by real-time RT-PCR.* Results*. EA shortened the stool efflux time and increased the dry fecal weight within 24 h in SCI rats. Daily rhythmicity of intestinal movements was unaffected by SCI. The expression of colonic* Per2* peaked at 20:00 and the nadir was observed at 8:00 in the SCI and sham groups. In the SCI+EA group, colonic* Per2* expression peaked at 12:00 and 20:00, and the nadir was observed at 8:00.* Conclusion*. SCI did not change the circadian rhythmicity of colonic* Per2* expression in rats, and daily intestinal movement rhythmicity was retained. EA changed the daily rhythmicity of intestinal movement and the circadian rhythmicity of colonic* Per2* expression in rats with SCI, increasing* Per2* expression shortly after EA treatment.

## 1. Introduction

Endogenous biological clocks in mammals are complex processes that include the central clock (also known as the master clock), located in the suprachiasmatic nucleus (SCN) of the hypothalamus, and the peripheral clocks (also known as the secondary clocks), located in peripheral organs such as the heart, liver, kidney, stomach, and intestine [1, 2]. These clocks regulate the 24 h body rhythmicity using transcription-translation negative feedback that consists of a series of clock genes and their products [2, 3]. The self-rhythm of the SCN master clock is affected by genetic factors and the environment [2, 4, 5]. The master clock affects the secondary clocks through nervous and humoral pathways, but the secondary clocks may also be affected by external factors such as stress, environment, diet, and injury [1].

Intestinal movements have a temporal rhythm in healthy mammals and are most active in the morning for diurnal mammals, while it is most active at nightfall for nocturnal mammals [4, 6, 7]. This cyclic rhythmic change of intestinal movements is regulated by the endogenous biological clocks [4]. The* Per2* gene is an important clock gene and is mainly expressed in epithelial cells and the myenteric plexus of the colon [8, 9]. Some studies revealed that the rhythmic changes of colonic motility were mainly regulated by peripheral clock genes including* Per2* [4, 8].

Spinal cord injury (SCI) may decouple the rhythmicity of the colon because of the loss of nervous signals from the central nervous system, leading to sensory and sphincter dysfunction [10–12]. In addition, severe SCI may also lead to impaired intestinal movement, manifesting as declined intestinal motility, slower peristalsis, and prolonged defecation time [10, 13]. Indeed, normal bowel movement is initiated by the central nervous system, which acts on the enteric nervous system and autonomic nervous system in multiple feedback manners [10, 13]. In the rat model of SCI, the action of the central nervous system is lost, but the enteric nervous system remains. Nevertheless, whether the biological clock without the action of the central nervous system still works properly is unknown. More than 33% of the patients with SCI may suffer from constipation, increased duration of defecation, abdominal distension, fecal incontinence, and other symptoms of intestinal dysfunction, affecting their quality of life [13–15]. Therefore, developing a therapeutic protocol that is efficient, simple, convenient, and inexpensive is of significance for improving the quality of life of patients with SCI.

Previous studies showed that acupuncture and electroacupuncture (EA) improved bowel dysfunction and colon motility in patients with SCI, without significant side effects [16–19]. Nevertheless, whether EA can regulate the biological clock of the intestine when deprived of the influence of the central nervous system is still unclear. Therefore, this study aimed to construct a rat model of severe SCI-induced intestinal dysfunction in order to evaluate the effects of EA on daily rhythmicity of intestinal movement and circadian rhythmicity of colonic* Per2* expression.

## 2. Materials and Methods

### 2.1. Animals and Grouping

Healthy adult specific pathogen-free grade Sprague-Dawley rats (*n* = 56; 28 males and 28 females; body weight of 200 ± 20 g) were purchased from Sippr BK Laboratory Animals Ltd. (Shanghai, China). The animals were adaptively fed to a body weight of 300 ± 20 g; they had free access to water and food and were kept at 23 ± 2°C and 50% humidity with a daily light/dark cycle of 12/12 h (daytime: 7:00–19:00). Postoperative animals were fed in separate cages.

The animals were randomized to three groups: (1) sham (sham operation, postoperative anti-infection agents, fixed in the prone position for 30 min starting at 11:00 daily 24 h after sham operation for 14 days); (2) SCI (modeling, postoperative anti-infection agents and nursing, fixed in the prone position for 30 min starting at 11:00 daily 24 h after modeling for 14 days); or (3) SCI+EA (modeling, postoperative anti-infection agents and nursing, EA treatment at bilateral Zusanli points for 30 min starting at 11:00 daily 24 h after modeling for 14 days).

Animal care was carried out in accordance with the* Instruction for Ethical Treatment of Animals* issued by the Ministry of Science and Technology, China, in 2006. We tried to minimize the number and suffering of the laboratory animals. All procedures and animal experiments were approved by the Animal Care and Use Committee of Nanjing University of Chinese Medicine (China).

### 2.2. Rat Models of Severe SCI

Rat models of severe SCI were established using the New York University Impactor [20]. Animals (*n* = 38) received intraperitoneal anesthesia with 10% chloral hydrate (300 mg/kg) and were fixed on the operating table in the prone position. After disinfection with iodophor, a median incision was made from T_10_ to T_13_. The skin was cut open and the superficial fascia was bluntly dissected to expose the spinous process and vertebral plates. The T_11_-T_12_ vertebral plates were removed with miniature mosquito forceps to generate a bone window and to expose the spinal cord. Then, the rats were fixed on a spinal impactor (New York University Impactor I, W.M. Keck Center for Collaborative Neuroscience Rutgers, State University of New Jersey, USA). The parameters were (1) weight of 10 g and (2) height of 60 mm. A severe spinal impact injury was made in the exposed spinal cord. A subdural hemorrhage at the impact point could be immediately observed and the rats showed lower limb twitching and tail lashing, followed by complete relaxation. After hemostasis, the layers were successively closed, and the wound was infiltrated with gentamicin. The successful modeling of SCI was determined using the improved BBB scale (improvement Basso, Beattie, Bresnahan locomotor rating scale) [21].

At three time points (before operation, 1 day after operation, and after 14 days of treatment), the animals were placed in an open space, and the motions of the hip, knee, and ankle joints, load bearing of hind limbs, ambulation, coordination of the four limbs, trunk stability, claws, and tail motions were observed independently and double blindly by two specialists. The motor functions of hind limbs were assessed using the improved BBB score. The score ranged from 0 to 21 points. A high score referred to a good motor function of the hind limbs. A score of 0 24 h after SCI represented successful modeling.

In the sham operation group (*n* = 18), the T_10_ to T_13_ spinous processes and vertebral plates were exposed without any treatment, and the wound was infiltrated with gentamicin and directly sutured 5 min later.

### 2.3. Postoperative Care

Rats were given intraperitoneal injections of 5000 U/kg of gentamicin from the day of operation and once daily for 15 days. Each day, rats' lower abdomen, perineum, and hind legs were cleaned, and the hind legs were passively exercised.

For postoperative assisted voiding, rats in the SCI group were held upright and their bladders were gently rubbed with the Crede top-down approach to assist with the discharge of urine, once every 12 h.

### 2.4. Electroacupuncture Treatment

Rats in the SCI+EA group were given EA treatment starting 24 h after successful modeling. Skin at bilateral Zusanli points (ST36, 3 cm below Dubi (ST 35), one finger width lateral to the anterior crest of the tibia), was routinely disinfected with iodophor, followed by direct punctures of 5 mm with disposable acupuncture needles (0.25*∗*13 mm, Suzhou Medical Instruments Co., Ltd., China) connected to an electroacupuncture apparatus (Hua Tuo electroacupuncture apparatus SDZ-II, Suzhou Medical Instruments Co., Ltd., China). EA settings were (1) disperse-dense wave; (2) 3 Hz/15 Hz; and (3) current intensity of 1-2 mA. The stimulation intensity was controlled to allow mild vibration of the needle handle and free vocalization of rats. This electric stimulation lasted 30 min, once daily, starting at 11:00, for 14 days.

In the sham and SCI groups, starting 24 h after operation, the rats were fixed for 30 min starting at 11:00 every day using the same method as in the SCI+EA group, for 14 days, but without EA.

### 2.5. Intestinal Transmission Function and Daily Rhythmicity of Intestinal Movement

Six rats were drawn from each group at 7:00 and 19:00 (*n* = 6/group/time point, *n* = 36) on day 14 of treatment. They had been fasted of food for 24 h but had received water ad libitum. They were given a gavage of 2 mL of 100 g/L activated charcoal suspension. The efflux time of the first black stool was recorded from the end of gavage administration of activated carbon.

After 14 days of treatment, the other six rats were drawn from each group, and their fecal pellets during daytime (7:00–19:00) and nighttime (19:00–7:00) were collected, dried, and weighed.

### 2.6. Hematoxylin and Eosin (H&E) Staining

Animals were sacrificed by cervical dislocation at day 16 after operation. The T_9–13_ spinal cord was exposed, and fresh spinal cord tissues (1.0 cm) were quickly harvested from a location centered on T_11_, followed by fixation with 4% paraformaldehyde. The abdomen was cut open, 1 cm of colon 1-2 cm away from the caecum was quickly sampled, cut open along the longitudinal axis, and the intestinal contents were washed with saline. The specimen was spread and fixed with 4% paraformaldehyde for >24 h. Tissues were dehydrated progressively with alcohol, made transparent with dimethylbenzene, embedded in paraffin, and sectioned at 4 *μ*m. Sections underwent H&E staining, followed by dehydration, air-drying, and sealing with neutral resins. Pathological changes of the spinal cord and colonic tissues were observed and photographed under a 40x light microscope (DMLS2, LEICA, Germany).

### 2.7. Real-Time RT-PCR

Three animals were drawn from each group at each time point (8:00, 12:00, 16:00, 20:00, 0:00, and 4:00) at day 16 after operation, from which the distal colonic tissues were obtained and cryopreserved in liquid nitrogen. RNA was extracted using the Total RNA Extraction Reagent (Vazyme Biotech Co., Ltd., Nanjing, China). RNA concentration and purity were determined using absorbance at 260 and 280 nm (A260/280). RNA (2.5 *μ*g) was reverse transcribed using HiScript™ Q RT SuperMix for qPCR (Vazyme Biotech Co., Ltd., Nanjing, China). Using GAPDH as an internal control, quantitative PCR was conducted with the dye method using the AceQ™ qPCR SYBR® Green Master Mix (Vazyme Biotech Co., Ltd.). The primers were synthesized by Vazyme. The primer sequences were* Per2*: forward 5′-CTG CCT ACC GCC ATC GAC-3′ and reverse 5′-TCT CCT CCT CTT TGG CTT CTG A-3′. GAPDH: forward 5′-GAG TCC ACT GGC GTC TTC A-3′ and reverse 5′-GGG GTG CTA AGC AGT TGG T-3′. The reaction conditions were 95°C (5 min), 95°C (10 s), 60°C (30 s), and 40 cycles. After PCR amplification, the Ct value was obtained by PCR software system and the results were calculated using the 2^−ΔΔCt^ method.

### 2.8. Immunohistochemistry

Colonic tissues were fixed with formaldehyde for more than 24 h, followed by routine paraffin embedding and sectioning (5 *μ*m). A section from each animal underwent immunohistochemistry according to the instructions provided by the MaxVision™ kit (Fuzhou Maixin Biotech Co., Ltd., China). A blank control group (primary antibody was replaced by PBS) was used. The* Per2* antibody was purchased from Molecular Tag. Inc. (USA) and was used at 1 : 200. Sections were observed under a light microscope and 200x images were taken.

### 2.9. Statistical Analysis

Statistical analyses were conducted using SPSS 16.0 (IBM, Armonk, NY, USA) and MATLAB (MathWorks, Natick, MA, USA). Data were expressed as mean ± standard deviation and analyzed using independent samples *t*-test, paired samples *t*-test, or one-way ANOVA with the least significant difference (LSD) (with homogeneity of variance) or Dunnett's T3 (no homogeneity of variance) post hoc test, as appropriate. Two-sided *P* values < 0.05 were considered statistically significant. The average colon* Per2* mRNA expression at six time points within 24 h was analyzed with the Cosinor analysis using MATLAB [22]. The cosine equation was *y* = *M* + *A* cos(*ωx* + Φ). The 7:00 time point was set as *x* = 0, while 8:00, 12:00, 16:00, 20:00, 0:00, and 4:00 were, respectively, corresponding to *x* = 1, 5, 9, 13, 17, and 21. *P* values < 0.05 were considered as indicating a circadian rhythmicity. By fitting the time series rhythm, we obtained mesor (*M*), amplitude (*A*), and acrophase (Φ), and we calculated the corresponding peak phase.

## 3. Results

### 3.1. Establishment of the Severe SCI Model

Before operation, the BBB score was 21 in all animals (*n* = 56). Among the 38 rats used for modeling, two died during surgery. In all 36 surviving rat models, the 24 h BBB score was 0, indicating model success. After EA treatment, the BBB score was 21 points in the sham group compared with 6–9 points in the SCI and SCI+EA groups.

In the SCI group, rats showed decreased bodyweight, activity, food intake, amount of feces, and urine retention. Two rats suffered from hematuria and four showed uracratia. All rats suffered from muscular dystrophy of the bilateral hind legs. In the SCI+EA group, rats showed milder urinary retention than those in the SCI group.

**Figure 1:**
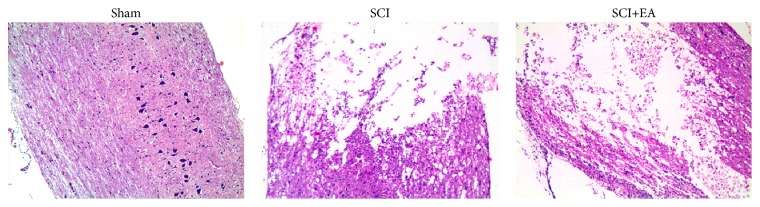
Pathological changes of spinal cord tissues in the spinal cord injury (SCI) rat model after electroacupuncture (EA) treatment for 14 days. Sham: sham operation, postoperative anti-infection agents, fixed in the prone position for 30 min starting at 11:00 daily 24 h after sham operation for 14 days; SCI: modeling, postoperative anti-infection agents and nursing, fixed in the prone position for 30 min starting at 11:00 daily 24 h after modeling for 14 days; SCI+EA: modeling, postoperative anti-infection agents and nursing, EA treatment at bilateral Zusanli points for 30 min starting at 11:00 daily 24 h after modeling for 14 days. Spinal cord tissues were stained by hematoxylin and eosin and observed under light microscopy (×40).

Rats in the sham group had intact spinal cord structure and normal morphology, with evenly arranged neuronal cells, and absence of necrosis and edema (Figure 1). Rats in the two SCI groups showed significant damage to the spinal cord tissues, structural disorder of local damaged tissues, necrosis of neuronal cells, abnormal tissue architecture, and edema (Figure 1).

### 3.2. Effect of EA on Intestinal Function in Rats with SCI

**Figure 2:**
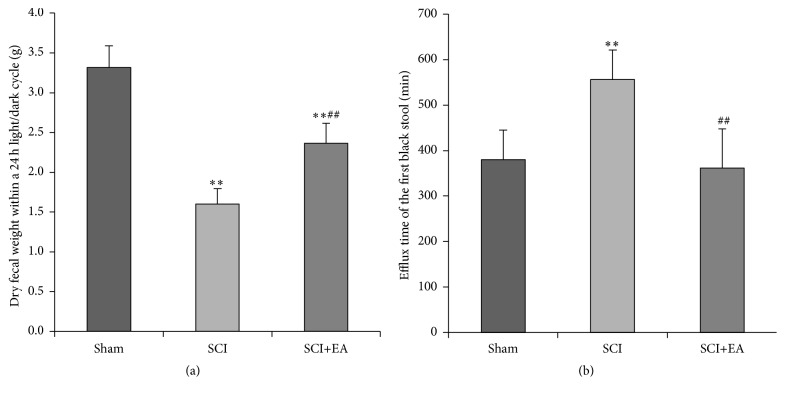
Effects of EA on intestinal transmission function in rats with SCI. (a) Dry fecal weight within a 24 h light/dark cycle. (b) Efflux time of the first black stool recorded from the end of gavage administration of activated carbon. Data are shown as mean ± standard deviation (SD) from *n* = 6/group for dry fecal weight or *n* = 12/group for efflux time. ^*∗∗*^ *P* < 0.01 versus sham; ^##^ *P* < 0.01 versus SCI.

Fifteen days after operation, rats in the sham group had normal stool and continuous discharge. Rats in the SCI group had dry and hard stool and single discharge of small granular fecal pellets; their 24 h dry fecal weight was reduced compared with that of the sham group (*P* < 0.01). Rats in the SCI+EA group also had small fecal pellets with a normal appearance and continuous discharge; their 24 h dry fecal weight within was larger than that of the SCI group (*P* < 0.01) (Figure 2(a)).

The efflux time of the first black stool in the SCI group was significantly longer than that in the sham group (*P* < 0.01), while EA shortened the efflux time of the first black stool compared with the SCI group (*P* < 0.01) (Figure 2(b)).

### 3.3. Effect of EA on Colonic Histopathology in Rats with SCI

**Figure 3:**
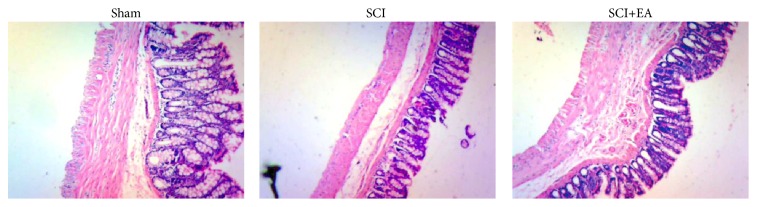
Effects of EA on colonic histopathology in rats with SCI. Colon tissues were stained by hematoxylin and eosin and observed under light microscopy (×40).

As shown in Figure 3, the SCI group showed intestinal wall atrophy, mucosal erosion, inflammatory cell infiltration, decreased proper glands, thinner muscular layer, and mild interstitial edema. The SCI+EA group presented mild atrophy of the muscular layer and proper glands and had colonic morphology similar to that of the sham group.

### 3.4. Effects of EA on Daily Rhythmicity of Intestinal Movement in Rats with SCI

**Figure 4:**
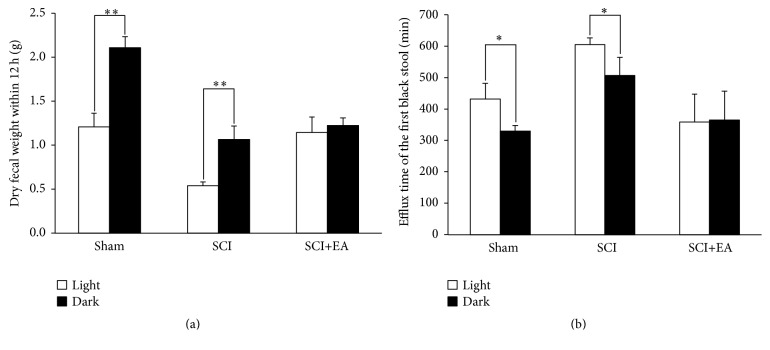
Effects of EA on daily rhythmicity of intestinal movement in rats with SCI. (a) Dry fecal weight and (b) efflux time of the first black stool recorded from the end of gavage administration of activated carbon during daytime (7:00–19:00) and nighttime (19:00–7:00) were measured, respectively. Data are shown as mean ± SD from *n* = 6/group for dry fecal weight or *n* = 6/group for efflux time. ^*∗*^ *P* < 0.05, ^*∗∗*^ *P* < 0.01 daytime versus nighttime.

Fifteen days after operation, the daytime dry fecal weight was smaller than during nighttime in the sham group (*P* < 0.01) (Figure 4(a)). The daily rhythmicity of intestinal movement in the SCI group was similar to that in the sham group, and the daytime dry fecal weight was smaller than during nighttime (*P* < 0.01), but the global dry fecal weight was smaller than that of the sham group. In the SCI+EA group, the daytime and nighttime dry fecal weights were similar (*P* > 0.05).

In the sham group, the efflux time of the daytime first black stool was significantly longer than during nighttime (*P* < 0.05), which was also observed in the SCI group (*P* < 0.05). In the SCI+EA group, the daytime and nighttime efflux times of the first black stool were similar (*P* > 0.05) (Figure 4(b)).

### 3.5. Effects of EA on Colonic* Per2* Expression Rhythmicity in Rats with SCI

**Figure 5:**
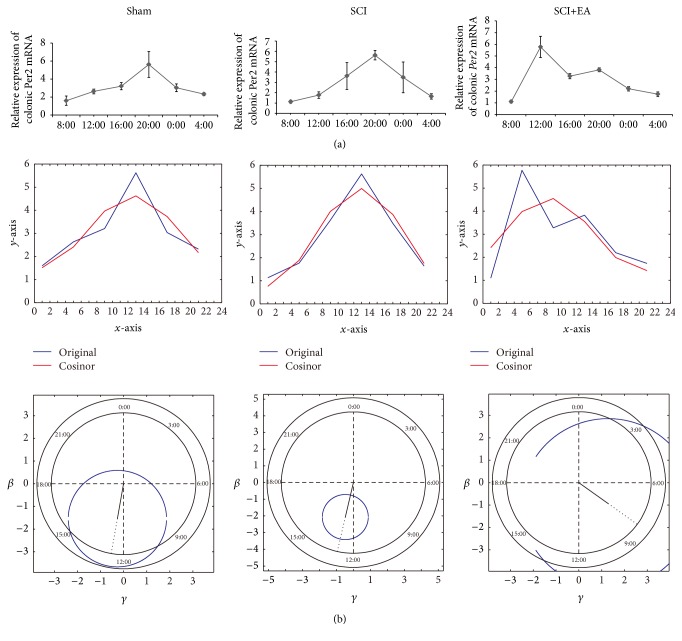
Effects of EA on circadian rhythmicity of colonic* Per2* gene expression in rats with SCI. (a) Relative expressions of colonic* Per2* mRNA in sham, SCI, and SCI+EA groups were determined by real-time RT-PCR. GAPDH was used as an inner control. Results are shown as mean ± SD (*n* = 3/group/time point). (b) Data were analyzed by Cosinor analysis using MATLAB. Top: *y*-axis: relative expression of colonic* Per2* mRNA, *x*-axis: Zeitgeber time, 0–12: daytime (7:00–19:00), 12–24: nighttime (19:00–7:00). The blue curve is the original average value (relative expression of colonic* Per2* mRNA); the red curve is the fitting cosine curve based on the average of the relative expression of colonic* Per2* mRNA. Bottom: *P* < 0.05 for the sham group and *P* < 0.01 for the SCI group, indicating a circadian rhythm in the sham and SCI groups. *P* > 0.05 for the SCI+EA group, indicating no circadian rhythm in the SCI+EA group. Peaks of the sham, SCI, and SCI+EA were at *x* = 12.66, *x* = 12.86, and *x* = 8.41, corresponding to 19:39, 19:52, and 15:24, respectively.

As shown in Figure 5(a), in the sham group, the relative expression of colonic* Per2* showed significant differences in time (*P* < 0.01 among the six time points) and temporal rhythmicity:* Per2* peaked at 20:00 (beginning of nighttime) and showed a nadir at 8:00 (beginning of daytime). The Cosinor analysis showed that the colonic* Per2* gene expression followed a circadian rhythm (*P* < 0.05), with *M* = 3.07, *A* = 1.56, and Φ = −3.31, and peak at *x* = 12.66 (corresponding to 19:39) (Figure 5(b)).

Similar results were observed in the SCI group (Figures 5(a) and 5(b)), that is, differences in time (*P* < 0.01 among the six time points) and temporal rhythmicity of* Per2* (peak at 20:00 and nadir at 8:00). The Cosinor analysis showed that the colonic* Per2* gene expression in the SCI group followed a circadian rhythm (*P* < 0.01), with *M* = 2.88, *A* = 2.12, and Φ = −3.37, and peak at *x* = 12.87 (corresponding to 19:52 h) (Figure 5(b)).

As shown in Figure 5(a), the relative expression of colonic* Per2* mRNA showed significant differences in time in the SCI+EA group, compared with that of the sham and SCI groups. EA treatment at the Zusanli point at 11:00–11:30 (daytime) daily for 14 days resulted in a peak of colonic* Per2* expression at 12:00 and in a higher expression level during the 12:00–16:00 period compared with the other time periods (4:00–8:00, 16:00–20:00 h, 20:00–0:00, and 0:00–4:00) in the SCI group. A second peak was observed at 20:00, but it was lower than the peak at 12:00. The nadir of colonic* Per2* expression was still observed at 8:00. The Cosinor analysis showed that the colonic* Per2* gene expression in the SCI+EA group did not follow a circadian rhythm (*P* > 0.05), with *M* = 2.98, *A* = 1.59, and Φ = −2.20, and peak at *x* = 8.41 (corresponding to 15:24 h) (Figure 5(b)).

### 3.6. Localization of* Per2* Protein in Colonic Tissues in Rats with SCI

**Figure 6:**
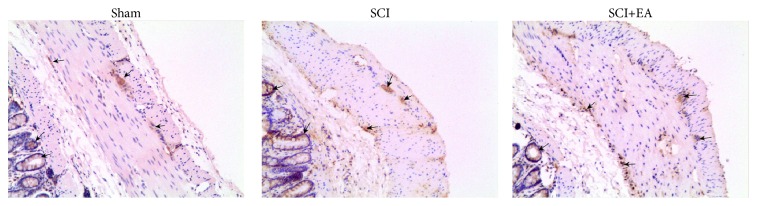
Localization of the* per2* protein in the colon tissue in rats with SCI. Colonic* Per2* protein expression was determined by immunohistochemistry (×200).

As shown in Figure 6, the Per2 protein was mainly observed in the colonic myenteric plexus and epithelial intestinal crypts (intestine glands). In addition, there was a small amount of* Per2* expression in the submucosal plexus of the colon.

## 4. Discussion

Bowel movements are subjected to the circadian cycle [23, 24], and colonic motility is regulated by the* Per2* gene [4, 8, 25]. SCI has been shown to affect the rhythmicity of the colon [10, 12] and EA may have an effect on the colonic circadian rhythm. Therefore, this study aimed to construct a rat model of severe SCI-induced intestinal dysfunction to observe the effects of EA on daily rhythmicity of intestinal movement and circadian rhythmicity of colonic* Per2* expression.

The results obtained in the present study showed that spinal cord histopathology in the SCI and SCI+EA groups was in accordance with the changes observed in severe SCI [26]. In addition, the rats from SCI group manifested prolonged defecation time, poor characteristics of fecal pellets, and small amount of feces, according to the findings of other authors [10, 13]. Colonic histopathology of rats in the SCI group showed bowel lumen expansion and pathological changes of the intestinal wall tissues. EA treatment tended to normalize the colonic morphology in rats with SCI.

In this study, rats in the sham group had a more active intestinal motility during nighttime than during daytime, presenting an obvious daily rhythmicity, as previously observed [4, 8, 9]. SCI did not affect daily rhythmicity of the intestinal mobility. Accordingly, the 24 h rhythmicity of the expression of colonic* Per2* in the SCI group was similar to that of the sham group. Previous studies demonstrated that the rhythmic expression of nocturnal rodent colonic clock genes (including Per2) was affected by daytime feeding and was independent of the central clock [8]. Meanwhile, rats receiving EA showed significant changes in rhythmicity of the colonic movements and in* Per2* expression. Therefore, these results suggest that EA treatment changed the daily rhythm of intestinal mobility in rats with SCI.

The Cosinor analysis showed that the circadian rhythm was maintained in the sham and SCI groups, but not in the SCI+EA group, suggesting that EA might promote colonic mobility, shorten the colonic transit time, increase dry fecal weight, and improve the corresponding symptoms of difficult defecation by regulating the* Per2* gene. Since the peak of* Per2* expression was observed shortly after EA in rats with SCI,* Per2* expression could be directly influenced by EA, changing the original daily rhythmicity of intestinal movement. Previous studies have shown that regular feeding was a strong factor involved in the rhythmicity of the colon [8, 27, 28]. The effect of EA on circadian rhythmicity of colonic* Per2* expression in rats with SCI was similar to the effect of regular food intake [8, 28, 29]. However, the regulatory effect of EA on intestinal dysfunction caused by SCI is more significant than the promoting effect of regular food intake on intestinal movement because human and animals have a limited food intake, and it is unlikely to promote intestinal movement by endless food intake. Moreover, an excessive intake of food may affect the normal intestinal function. Thus, an appropriate dietary management is needed for patients with SCI [30–32].

Based on histopathological examination, the extent of damage to the spinal cord was comparable between the SCI and SCI+EA groups, suggesting that EA at the Zusanli point had no effect on the repair of SCI. The Zusanli point is a commonly used acupoint for the treatment of gastrointestinal diseases [33]. Skin and muscles in the Zusanli area are dominated by superficial and deep peroneal nerves, which are fired from the L4-S2 segments of the spinal cord. Some studies revealed that EA treatment at the Zusanli point could regulate the electrical activity and mechanical energy of the smooth muscles through cholinergic and nitrergic neurotransmitter signals in the enteric nervous system and other gastrointestinal hormones [34–36].

Immunohistochemistry revealed that the colonic* Per2* protein was mainly located in the intestinal myenteric plexus and epithelial intestinal crypt (glands of the large intestine) and that there was a small amount of expression in the submucosal plexus of the colon, which was consistent with previous reports [8, 9]. The brain SCN clock contains multiple central clock genes including* Per2* and regulates peripheral organs through affecting the release of various neurotransmitters and hormones. Similarly, studies found that a variety of colonic clock genes including* Per2* could affect intestinal movements through regulating release of part of the excitatory and inhibitive neurotransmitters of the enteric nervous endings [4, 8].

## 5. Conclusion

SCI did not change the circadian rhythmicity of colonic* Per2* expression in rats, in which daily intestinal movement rhythmicity was retained. EA changed the daily rhythmicity of intestinal movement and the circadian rhythmicity of colonic* Per2* expression in rats with SCI, increasing* Per2* expression shortly after EA treatment. EA could be used to improve bowel function after SCI.
